# P-1910. Validation of a KAP Instrument on Physician’s Prevention and Management of Arboviral Diseases: Design and validation in Venezuela

**DOI:** 10.1093/ofid/ofaf695.2079

**Published:** 2026-01-11

**Authors:** Oriana A Regalado-Gutiérrez, David Forero-Peña, Fhabian S Carrión-Nessi, Maya A Ocanto-Yztúriz, Luz M Ríos-Di Ciaccio, Andreina H Rondón-Pérez, Iriangel S Hidalgo-García

**Affiliations:** Central University of Venezuela, Caracas, Miranda, Venezuela; Biomedical Research and Therapeutic Vaccines Institute, Ciudad Bolívar, Venezuela, Caracas, Distrito Federal, Venezuela; Biomedical Research and Therapeutic Vaccines Institute, Ciudad Bolívar, Venezuela, Caracas, Distrito Federal, Venezuela; Central University of Venezuela, Caracas, Miranda, Venezuela; Central University of Venezuela, Caracas, Miranda, Venezuela; Central University of Venezuela, Caracas, Miranda, Venezuela; Central University of Venezuela, Caracas, Miranda, Venezuela

## Abstract

**Background:**

The incidence of arboviral diseases has progressively increased in the Americas, assigning medical personnel a crucial role in the diagnosis and management of these infections. Although PAHO guidelines exist, a validated instrument to evaluate physician performance in this area remains unavailable. This study validates an instrument designed to assess medical personnel's clinical approach concerning arbovirus management in Venezuela.

**Methods:**

Following PAHO recommendations, an online survey targeting physicians in Venezuela compared frontline and non-frontline workers. Two-step cluster analysis was used to classify KAP dimensions. The instrument’s internal consistency was assessed using Cronbach's alpha and Kuder-Richardson-20. Correlations between KAP dimensions were explored using Spearman's coefficient.

**Results:**

A total of 127 physicians from 18 states participated (median age 32 years, IQR 29-41). Participants were predominantly frontline physicians (68%), and the sample included general practitioners (35.4%) and specialists (37%). Regarding training, only one-third reported recent training in arboviruses, and about half in notifiable diseases report. A significantly higher proportion of frontline physicians had received this training (*p*=0.002 and *p*=0.031, respectively). Knowledge levels were predominantly intermediate (47.2%) or low (30.7%), with no significant differences observed between groups (*p*=0.374). Correlation analysis revealed a positive association between high knowledge and positive attitudes (*r*_s_=0.195, *p*=0.028), and between these and appropriate practices (*r*_s_=0.197, *p*=0.027). Internal consistency showed good performance in the practices dimension (α=0.701), and room for improvement in attitudes (α=0.507) and knowledge (KR-20=0.495).

**Conclusion:**

This instrument provides a valuable resource for strengthening continuing medical education by identifying key gaps that can guide targeted training interventions to improve the diagnosis and management of these public health threats. Additionally, it can function as an initial prototype for use in other parts of the region.

**Disclosures:**

All Authors: No reported disclosures

